# Inflammatory bowel disease serologies in ankylosing spondylitis patients: a pilot study

**DOI:** 10.1186/ar2866

**Published:** 2009-11-23

**Authors:** Matthew L Mundwiler, Ling Mei, Carol J Landers, John D Reveille, Stephan Targan, Michael H Weisman

**Affiliations:** 1 Rockford Orthopedic Associates, 324 Roxbury Rd, Rockford, IL 61107, USA; 2 Center for Inflammatory Bowel Disease, Cedars-Sinai Medical Center, 8700 Beverly Blvd, Los Angeles, CA 90048, USA; 3 Rheumatology, University of Texas Health Sciences Center, 6431 Fannin Rm 5270, Houston, TX 77030, USA

## Abstract

**Introduction:**

Ankylosing spondylitis (AS) and inflammatory bowel disease (IBD) share similarities and are classified as spondyloarthropathies. In IBD, anti-*Saccharomyces cerevisiae *antibody (ASCA), anti-I2 (associated with anti-*Pseudomonas *activity), anti-*Escherichia coli *outer membrane porin C (anti-OmpC), anti-flagellin (anti-CBir1), and antineutrophil cytoplasmic antibodies (ANCA) possess clinical significance. Because of the overlap between the two conditions, a pilot study was designed to compare the frequency of these antibodies in AS patients compared to normal controls.

**Methods:**

Serum stored from 80 AS patients and 80 control subjects was available for analysis. ASCA, anti-I2, anti-OmpC, anti-CBir1, and ANCA studies were completed on all serum samples using Enzyme-Linked Immunosorbent Assay (ELISA) methodology. The following analyses were performed: comparison of positivity based on the established values in IBD, median values, the number of subjects in each serology in the 4^th ^quartile of a normal distribution, and the mean quartile sum of all the antibodies.

**Results:**

There was no difference in positivity rates between AS and control groups with the established IBD values. The median anti-I2 response was significantly higher in AS than in controls (11.78 vs 7.86, p = 0.017). Significantly more AS patients had quartile scores of 4 for the following antibody responses: ASCA IgG (26% vs 13%, p = 0.016, OR = 2.49, CI 1.168 - 5.313), ASCA IgG and IgA (27% vs 12%, p = 0.006, OR = 2.9, CI: 1.342 - 6.264), and anti - I2 (25% vs 14%, p = 0.0424, OR = 2.15, CI: 1.018 - 4.538). The mean quartile sum of the antibody responses was elevated in AS patients when ANCA was excluded (10.526 vs 9.519, p = 0.03). When ANCA was included, this difference lost significance.

**Conclusions:**

The data from this pilot study points towards mucosal dysregulation as an important pathway in AS. We were able to demonstrate that anti-I2 could play a pathologic role in AS. The elevated mean total antibody response being significant only with ANCA exclusion is consistent with the histopathological evidence that intestinal inflammation in AS is similar to Crohn's disease. To better define the roles of these antibodies in AS, larger studies with more precisely defined patient characteristics are required.

## Introduction

Ankylosing spondylitis (AS), an inflammatory disease of unknown etiology, is characterized by a progressive and destructive inflammatory arthritis of the spine, peripheral joints, and entheses, sometimes with extra-spinal manifestations. A recent review of the epidemiology of AS in the USA indicates that the prevalence is 0.52%, with the prevalence of overall spondyloarthritides ranging from 0.34 to 1.31% depending on diagnostic criteria [1]. The distinguishing pathologic aspect of AS is the presence of reactive bone growth forming syndesmophytes, and these features are associated with ankylosis of articulating structures causing limited mobility, abnormal posture, and increased fracture risk [2]. Extra spinal disease secondary to AS includes uveitis, cardiac valve dysfunction, renal disease due to secondary amyloidosis, and microscopic intestinal inflammation [3].

AS is considered to be part of a larger group of disorders known as the spondyloarthritides that includes inflammatory bowel disease (IBD), which is usually classified as Crohn's disease (CD) or ulcerative colitis (UC) depending on the inflammatory pattern. Although intestinal inflammation predominates in patients with IBD, some patients possess axial arthritis affecting the spine and sacroiliac joints that can be indistinguishable from AS. For example, it has been observed that 10 to 20% of patients with IBD have sacroiliac changes and 7 to 12% of patients have a concomitant diagnosis of AS, a rate approximately 10 times that of the non-IBD population [4]. To further support this relation between AS and IBD, investigators have noted that a significant percentage of patients with AS also have intestinal inflammation. Of patients with AS, 26% have histopathological intestinal inflammation consistent with CD, and 6.5 to 10% possess a diagnosis of IBD [3]. Furthermore, similar genetic predisposition is shared by these two diseases. Both patients with AS and IBD have an increased prevalence of human leukocyte antigen (HLA)-B27, 85% and 33%, respectively [4], and, more recently, the IL23R gene has been shown to be associated with both IBD and AS [5,6].

CD is associated with a selected loss of tolerance to commensal microbiota as evidenced by circulating antibodies to a subset of microbial antigens [7], including anti-*Saccharomyces cerevisiae *antibody (ASCA), anti-I2 (associated with anti-*Pseudomonal *activity), anti-*Escherichia coli *outer membrane porin C (anti-OmpC), anti-flagellin (anti-CBir1), and antineutrophil cytoplasmic antibodies (ANCA). Previous studies have shown evidence of loss of tolerance by exhibiting elevated ASCA IgA levels in AS patients [8,9]. This finding, however, is not always replicated [10].

Because of the IBD-like mucosal changes that occur in a substantial percentage of AS patients, we hypothesized that serologic activity normally used to detect loss of tolerance to enteric antigens related to mucosal dysregulation in IBD will be detectable in levels above normal controls in AS patients. To determine whether AS and CD or IBD have similar abnormalities in loss of mucosal tolerance, we tested antibody reactivity to these microbial antigens in a pilot exploratory study with a limited number of patients to see if a signal indicating further, more comprehensive testing would be warranted.

## Materials and methods

### Patient selection

Eighty patients were randomly selected from the Prospective Outcomes in Ankylosing Spondylitis (PSOAS) cohort, an AS cohort previously assembled from patients from Cedars-Sinai Medical Center, University of Texas at Houston, National Institutes of Health, and the University of California at San Francisco [11,12]. All patients are 18 years of age or older and have supplied written consent to be a part of the cohort and related studies. These patients meet the modified New York criteria for AS: low back pain and stiffness for more than three months which improves with exercise but is not relieved by rest OR limitation of the lumbar spine in the sagittal and frontal planes OR limitation of chest expansion relative to normal values corrected for age and sex AND erosions and ankylosis of at least one sacro-iliac joint or sclerosis in the bilateral sacro-iliac joints [13]. If a selected sample was from a patient with IBD by self-report, another sample was selected. Eighty control patients whose serum was already collected and stored at -70°F in Houston, Texas, were used. These patients are 18 years of age or older, have supplied written consent, and are free from rheumatic disease. The size of the group was determined to power the study to detect a statistically significant difference if the frequency of positivity for any single serology was 15% in the AS group and 5% in the control group. The study was approved by the Institutional Review Board at all participating centers.

### Laboratory methods

The samples were collected and re-labeled to blind laboratory personnel to their source. All serologic tests were run on the samples at the same time in an effort to decrease variance. The basic laboratory methods for the determination of each serology have been described elsewhere [7,14]. Sera was tested by ELISA for the five individual serologies: ANCA, ASCA IgG and IgA, Anti-I2, Anti-OmpC, and Anti-CBir1. The results of each assay are expressed in ELISA units and reflect antibody activity except for ASCA. ASCA results were log transformed prior to quartile distribution.

After the testing was complete and the antibody activity levels determined, the results were unblinded to determine their origin. The groups were compared using the following statistical methodologies.

### Statistical methods

In the first analysis, chi-squared testing was performed to compare the positivity rates for each separate serology in AS patients versus controls. The reference values usually applied to IBD were used (Table 1). Next, the Wilcoxin Rank test was performed to compare the median quantitative antibody level between cases and controls.

**Table 1:** Reference values for antibodies tested and their values in Crohn's disease

Antibody	Positive cutoff reference value	Median value in CD, Range	Percent positive in CD
ASCA IgG	20 EU	26.9, 0-415.2	38.1%
ASCA IgA	40 EU	12.5, 0-164	38.1%
Anti-I2	30 EU	18.9, 0-324.1	46.8%
Anti-OmpC	23 EU	15.2, 0-296.4	31.7%
Anti-CBir1	30 EU	26.1, 0-279.7	49%
ANCA	30 EU	1.4-277.7*	74%*

The positive cutoff value is two standard deviations above the mean value in healthy controls. *These values apply to ulcerative colitis as opposed to crohn's disease.

ANCA = antineutrophil cytoplasmic antibodies; anti-CBir1 = anti-flagellin; anti-OmpC = anti-*Escherichia coli *outer membrane porin C; ASCA = Anti-*Saccharomyces cerevisiae *antibody; CD = Crohn's disease; EU = ELISA units.

For the next analysis, a distribution of the magnitude of each serologic response for all subjects was divided into four quartiles. A score of 1 to 4 was assigned on the basis of the designated quartile (<25% = 1; 25 to 50% = 2; 51 to 74% = 3; 75 to 100% = 4). Chi-squared testing was performed to compare the percentage of cases and controls with a score of 4 versus 1, 2, or 3. Chi-squared testing was repeated to compare cases and controls with a score of 3 and 4 versus 1 and 2. To determine the significance of increasing odds ratio (OR) for AS with increasing quartile score for each antibody, the Cochran-Armitage test was performed. For the OR calculations, the minimum quartile score of 1 was set as the baseline, that is, an OR of 1.0. In the final analysis, student t-test was performed to compare the mean quartile sum between cases and controls. All the analyses were performed by SAS computer software (version 9.13; SAS Institute, Inc., Cary, NC, USA).

## Results

Seventy nine patients each from both the AS and normal control groups yielded results suitable for analysis. The demographic information for each group is in Table 2. When the reference ranges for IBD are applied, there is no difference in the positivity rates between AS patients and controls (Table 3).

**Table 2:** Demographic information for AS patients and controls

	AS patients	Controls
Age in years (mean, range)	36.5, 19-61	30.8, 18-104
Disease duration in years (mean, range)	10.3, 0.12-43	Not applicable
Male (%)	62%	42%
HLA B27 (%)	84%	Not available
Ethnicity	Caucasian 80% African American 2.5% Asian 3.8% Hispanic 3.8% Caucasian and Hispanic 8.8% African American and Caucasian 1%	Caucasian 100%

HLA-B27 status for the control group was unavailable.

AS = ankylosing spondylitis; HLA = human leukocyte antigen.

**Table 3:** Positivity rates of serologies in AS patients and controls

Antibody	Positive AS patients	Positive controls	*P *value
IgA ASCA	1.3% (1)	0% (0)	1.0
IgG ASCA	3.8% (3)	1.3% (1)	0.62
Anti-I2	12.7% (10)	5.1% (4)	0.09
Anti-OmpC	3.8% (3)	2.5% (2)	1.0
Anti-CBir1	17.7% (14)	11.4% (9)	0.25
ANCA	6.35 (5)	5.1% (4)	1.0

Normal cut-offs normally utilized in IBD applied here.

ANCA = antineutrophil cytoplasmic antibodies; anti-CBir1 = anti-flagellin; anti-OmpC = anti-*Escherichia coli *outer membrane porin C; AS = ankylosing spondylitis; ASCA = Anti-*Saccharomyces cerevisiae *antibody.

However, when comparing the median level of the antibody response of each serology in AS patients versus controls (Table 4), anti-I2 levels were significantly elevated in AS patients (11.78 vs. 7.86, *P *= 0.017).

**Table 4:** Median value of titers in AS patients versus controls and results of Z-testing

Antibody	Median value of titer in AS patients	Median value of titer in control patients	*P *value
IgA ASCA	1.24	1.10	0.28
IgG ASCA	4.70	4.05	0.29
***Anti-I2***	***11.78***	***7.86***	***0.0172***
Anti-OmpC	8.61	7.70	0.30
Anti-CBir1	16.26	15.05	0.63
ANCA	11.6	12.1	0.65

The median value of Anti-I2 in AS patients is significantly increased when compared to controls (shown in bold and italics).

ANCA = antineutrophil cytoplasmic antibodies; anti-CBir1 = anti-flagellin; anti-OmpC = anti-*Escherichia coli *outer membrane porin C; AS = ankylosing spondylitis; ASCA = Anti-*Saccharomyces cerevisiae *antibody.

**Figure 1:**
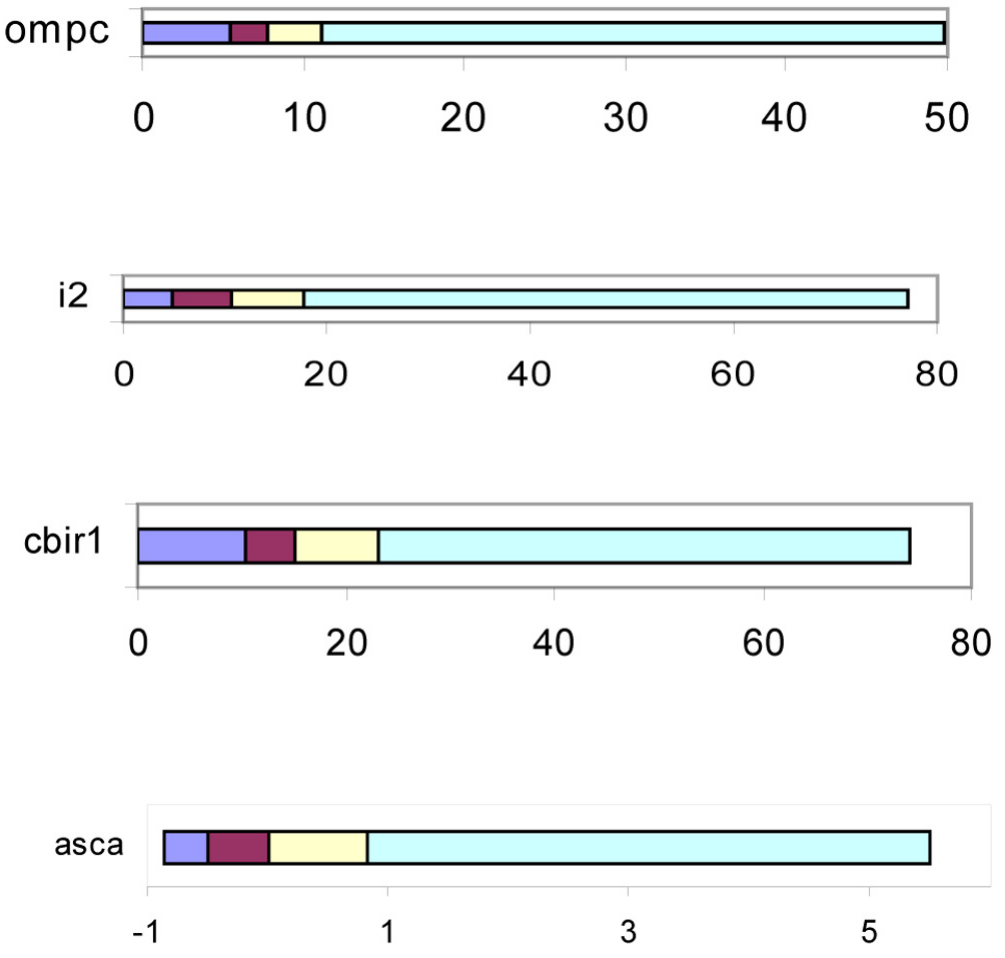
Quartile distributions for anti-OmpC, anti-I2, anti-Cbir1, and ASCA IgG and IgA. Each colored section of the bar represents approximately 40 patients from all the subjects in the study, both ankylosing spondylitis patients and controls. anti-CBir1 = anti-flagellin; anti-OmpC = anti-*Escherichia coli *outer membrane porin C; ASCA = Anti-*Saccharomyces cerevisiae *antibody.

**Figure 2:**
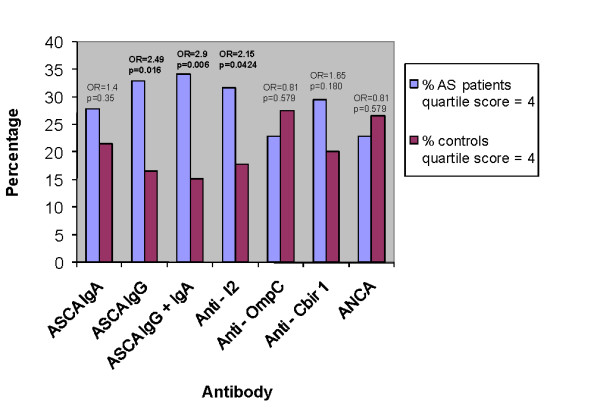
AS patients versus controls for each serology with a quartile score of 4. Significant results are in bold. Confidence intervals for significant odds ratios are: ASCA IgG (1.168, 5.313), ASCA IgG and IgA (1.342, 6.264), and anti-I2 (1.018, 4.538). anti-CBir1 = anti-flagellin; anti-OmpC = anti-*Escherichia coli *outer membrane porin C; ASCA = Anti-*Saccharomyces cerevisiae *antibody.

Given the suggestion that there were differences between controls and AS subjects in the level of antibody responses, we performed a more extensive analysis using approaches that have been used in evaluating these responses in IBD [7,14-16]. The antibody results of the patients and controls were distributed and divided into quartiles (Figure 1). Subjects with AS were more likely to have a quartile score of 4 (upmost quartile) for anti-I2, ASCA IgG, and total ASCA (IgG + IgA) (Figure 2). The analysis was repeated to determine if there were serologies that had higher numbers of AS patients with a quartile score of 3 and 4 combined. Only anti-I2 yielded a significant difference (62% AS patients vs 39% controls, OR = 2.67, confidence interval (CI) = 1.403 to 5.072, *P *= 0.003).

**Figure 3:**
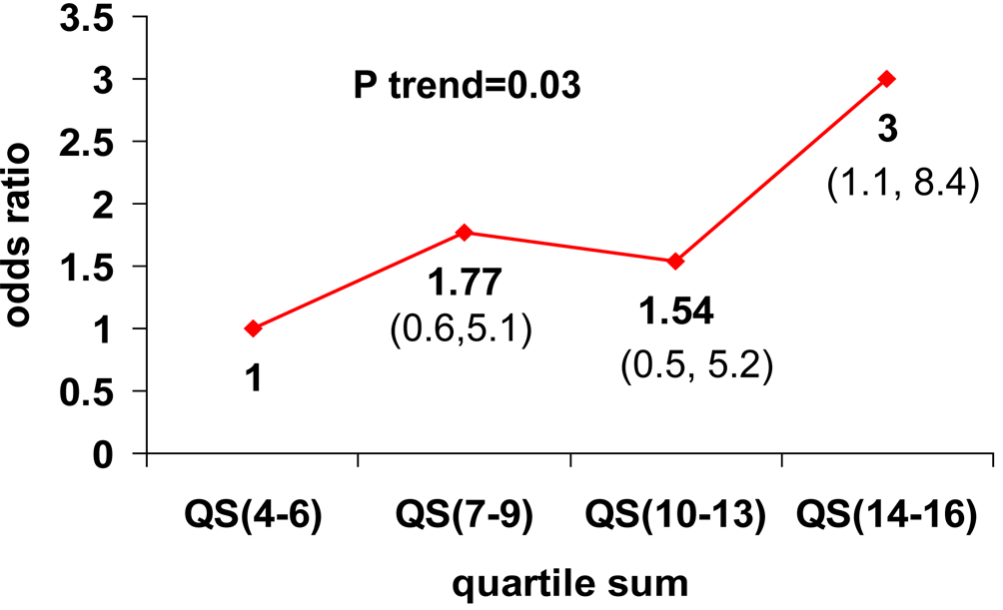
Overall trend of odds ratios showing a higher percentage of AS patients in the higher quartile sums. The overall trend when comparing all values was significant (*P *= 0.03). The odds ratio with confidence intervals in parentheses for each data point are labeled.

Finally, we compared the quartile sum of all serologies in AS versus controls. The level of each antibody was normalized by dividing the cohort into quartiles. The overall level of response to the antibodies was measured by summing the individual quartiles to yield a quartile sum. The elevated mean quartile sum seen in AS patients was higher than controls when ANCA was excluded (10.526 vs 9.519, *P *= 0.03). When ANCA was included, the difference in mean quartile sum lost significance. Furthermore, the number of AS patients in each quartile group increased as the quartile sum increased as shown by the increasing ORs (group 4-6 (reference groups) vs. 7-9 vs. 10-13 vs. 14-16: 1 vs. 1.77 vs. 1.54 vs. 3.0, *P *trend = 0.03). The summary of this comparison is shown in Figure 3.

## Discussion

In this pilot study, our results indicate that mucosal dysregulation demonstrated by elevated antibody responses to CD-related enteric microbial antigens is present in a cohort of AS patients. Using a quartile analysis method that allows for a more sensitive evaluation of levels of these CD-related microbial antigens when compared with disease control cohorts, we detected an increased individual antibody response to I2 and ASCA, as well as a higher response to the combination of antibodies in patients compared with controls.

The quartile analysis methodology has yielded significant results in IBD studies utilizing these serologies as indicators of mucosal dysregulation. Demonstrating the strength of this analysis to determine differences in non-IBD but related individuals was previously demonstrated by Devlin and colleagues [17]. Using quartile sums, the level of antibody responses in CD patients was related to the number of NOD2 variants (0-2) in a given individual. The same analysis was then repeated using unaffected family members of CD patients. Although some family members have elevated levels as defined by cut offs used to differentiated CD from normal controls, overall the levels were much lower than CD patients. However, when the quartile sums were recalculated using the levels of antibodies within entire unaffected family members as the cohort and not CD, the same relation of numbers of NOD2 variants to quartile sums was seen as that in the CD cohort. An important concept from these analyses is that the current laboratory methods are tailored to have optimal sensitivity and specificity for patients with CD. However, there are no established reference ranges or methods for detecting these antibody values in AS subjects. In the future, it may be possible to develop a method to study the AS population at large using appropriate controls.

In addition, we observed that the sum of antibody activity is higher in AS patients compared with controls if ANCA is excluded. In our study, ANCA levels in AS patients were not significantly elevated when compared with controls. In IBD studies where these issues are addressed, pANCA (perinuclear ANCA) positivity is most typically associated with UC as opposed to CD [18]. This finding from our study is consistent with clinical observations that intestinal inflammation associated with AS more closely resembles the phenotype of CD rather than UC [4]. Thus, our pattern of serological detection is consistent with what is hypothesized about the relation between AS and CD.

In IBD, detecting the loss of tolerance to certain antigens has diagnostic and prognostic significance. For example, determining the pANCA and ASCA status of IBD patients helps determine if the patient's phenotype will be more consistent with CD or UC with a high specificity [18]. Furthermore, a pANCA antibody response in a CD patient signifies a predilection towards left-sided large intestine involvement [19]. Outcome studies have revealed that anti-I2 activity is associated with increased likelihood of fibrostenosing CD, that anti-OmpC activity is associated with increased likelihood of perforation, and patients with loss of tolerance to multiple antigens are more likely to undergo small bowel surgery [15]. Additionally, anti-CBir1 activity is associated with penetrating, fibrostenosing disease [16]. Further detection of these serologies have also aided in revealing that mucosal dysregulation exists in unaffected relatives who carry genetic markers for IBD in comparison with controls who do not [17,20].

With these findings reported herein, it is our hope that we can make similar pathologic and clinical associations with the presence of mucosal dysregulation and AS. Although the cause of AS is still unknown, associating mucosal dysregulation with disease onset of AS would bring us closer to potentially discovering the trigger that initiates the disease phenotype called AS. Further, as in IBD, these serologies may help provide clinical information in AS that could indicate which AS patients are more likely to have intestinal inflammation or even develop overt IBD. They may help determine patients who are more likely to have aggressive disease, different phenotypic patterns of ankylosis, or response to biologics.

There are obvious limitations to interpreting the results from this pilot study. First, our sample size is small. A larger study is required to more precisely determine the true prevalence as well as the significance of these serologies in AS. Furthermore, these patients are a random sampling from an AS cohort who are only characterized by the absence of IBD. Further studies will serve to place patients in a clinical context providing disease associations with disease onset and duration, clinical characteristics, disease severity, and treatment response.

## Conclusions

In conclusion, this pilot study provides evidence that mucosal dysregulation could play a significant role in AS. To better define the significance of the findings, larger studies with more precisely defined patient characteristics are required.

## Abbreviations

ANCA: antineutrophil cytoplasmic antibodies; anti-CBir1: anti-flagellin; anti-OmpC: anti-*Escherichia coli *outer membrane porin C; AS: ankylosing spondylitis; ASCA: anti-*Saccharomyces cerevisiae *antibody; CD: Crohn's disease; CI: confidence interval; ELISA: enzyme-linked immunosorbent assay; HLA: human leukocyte antigen; IBD: inflammatory bowel disease; IL-23R: interleukin-23 receptor; OR: odds ratio; PSOAS: Prospective Outcomes in Ankylosing Spondylitis; UC: ulcerative colitis.

## Competing interests

The authors declare that they have no competing interests.

## Authors' contributions

MLM submitted the study for approval, helped determine the analysis strategy, and wrote the manuscript. LM performed the statistical operations, constructed figures, and reviewed the manuscript. CL coordinated and oversaw all laboratory work. JDR provided control samples and input on the manuscript. ST provided the laboratory resources, gave extensive input on study design, and was instrumental in interpreting the results. MHW managed the AS cohort, initiated the study, oversaw analysis, and approved the final manuscript.
